# Pachydermoperiostosis Presenting With Gynecomastia and Low Insulin-Like Growth Factor-1 Levels: A Diagnostic Challenge

**DOI:** 10.7759/cureus.99502

**Published:** 2025-12-17

**Authors:** Jonnalagadda Amith Priyansu, Rakesh U. K., Sridhar Amalakanti, Mohammad Asif, Sai V Chitturi

**Affiliations:** 1 General Medicine, All India Institute of Medical Sciences, Mangalagiri, Mangalagiri, IND; 2 Medicine, Guntur Medical College, Guntur, IND; 3 Internal Medicine, St. Martinus University Faculty of Medicine, Willemstad, CUW

**Keywords:** digital clubbing, pachydermoperiostosis, periostosis, primary hypertrophic osteoarthropathy, touraine-solente-golé syndrome

## Abstract

Pachydermoperiostosis (PDP), or primary hypertrophic osteoarthropathy, is a rare genetic disorder characterized by digital clubbing, skin thickening, and periostosis. Due to its overlapping clinical features with secondary hypertrophic osteoarthropathy, it often poses a diagnostic challenge. We report a case of a 20-year-old male patient who presented with progressive digital clubbing, joint pain, and coarse facial features over four years. The patient also exhibited gynecomastia, an uncommon feature in PDP. Despite no history of underlying pulmonary, cardiac, or gastrointestinal disease, his symptoms persisted. Hormonal evaluation revealed low insulin-like growth factor-1 (IGF-1) levels with normal pituitary imaging, effectively excluding acromegaly. Genetic and radiographic studies were not performed due to logistical constraints. Diagnosis was established through clinical evaluation and exclusion of secondary causes. The patient was managed symptomatically with nonsteroidal anti-inflammatory drugs (NSAIDs) and lifestyle modifications, resulting in partial relief of joint pain and stiffness. This case highlights the importance of recognizing PDP in young patients presenting with clubbing and skin changes, especially in the absence of secondary causes. Early diagnosis and multidisciplinary management are essential to prevent unnecessary investigations and improve patient outcomes.

## Introduction

Pachydermoperiostosis (PDP), also known as primary hypertrophic osteoarthropathy or Touraine-Solente-Golé syndrome, is a rare genetic disorder characterized by the triad of digital clubbing, periostosis, and pachydermia [1]. It is caused by mutations in the *HPGD* or *SLCO2A1* genes, which disrupt prostaglandin metabolism [2]. PDP is often misdiagnosed as secondary hypertrophic osteoarthropathy, leading to unnecessary investigations [3].

PDP typically manifests during adolescence or early adulthood, with a male predominance (male-to-female ratio ≈ 7:1). The disease progresses slowly over several years before stabilizing [4]. It follows an autosomal dominant inheritance pattern with variable penetrance, although sporadic cases are also reported. Clinically, patients may exhibit digital clubbing, periostosis, pachydermia, hyperhidrosis, and arthralgia. Management is primarily symptomatic, involving nonsteroidal anti-inflammatory drugs (NSAIDs), bisphosphonates, and dermatologic care for skin changes [5]. Differential diagnoses include acromegaly, thyroid acropachy, and secondary hypertrophic osteoarthropathy [6]. In rare cases, hormonal abnormalities such as gynecomastia and altered insulin-like growth factor-1 (IGF-1) levels have been described, adding complexity to diagnosis [7,8].

In this context, we present the case of a 20-year-old male patient with PDP presenting with classic clinical features along with gynecomastia, a rare association, and discuss the diagnostic challenges and management.

## Case presentation

A 20-year-old male patient presented to our outpatient department with a five-year history of gradually worsening symptoms that had significantly impacted his daily life. He first noticed thickening of the skin on his face and scalp, accompanied by an unusual enlargement of his hands and feet. Over time, these changes became more pronounced, and he began experiencing persistent joint pain, particularly in his wrists and ankles. He had no history of fever, weight loss, or respiratory complaints, which often accompany secondary causes of similar clinical presentations. 

**Figure 1:**
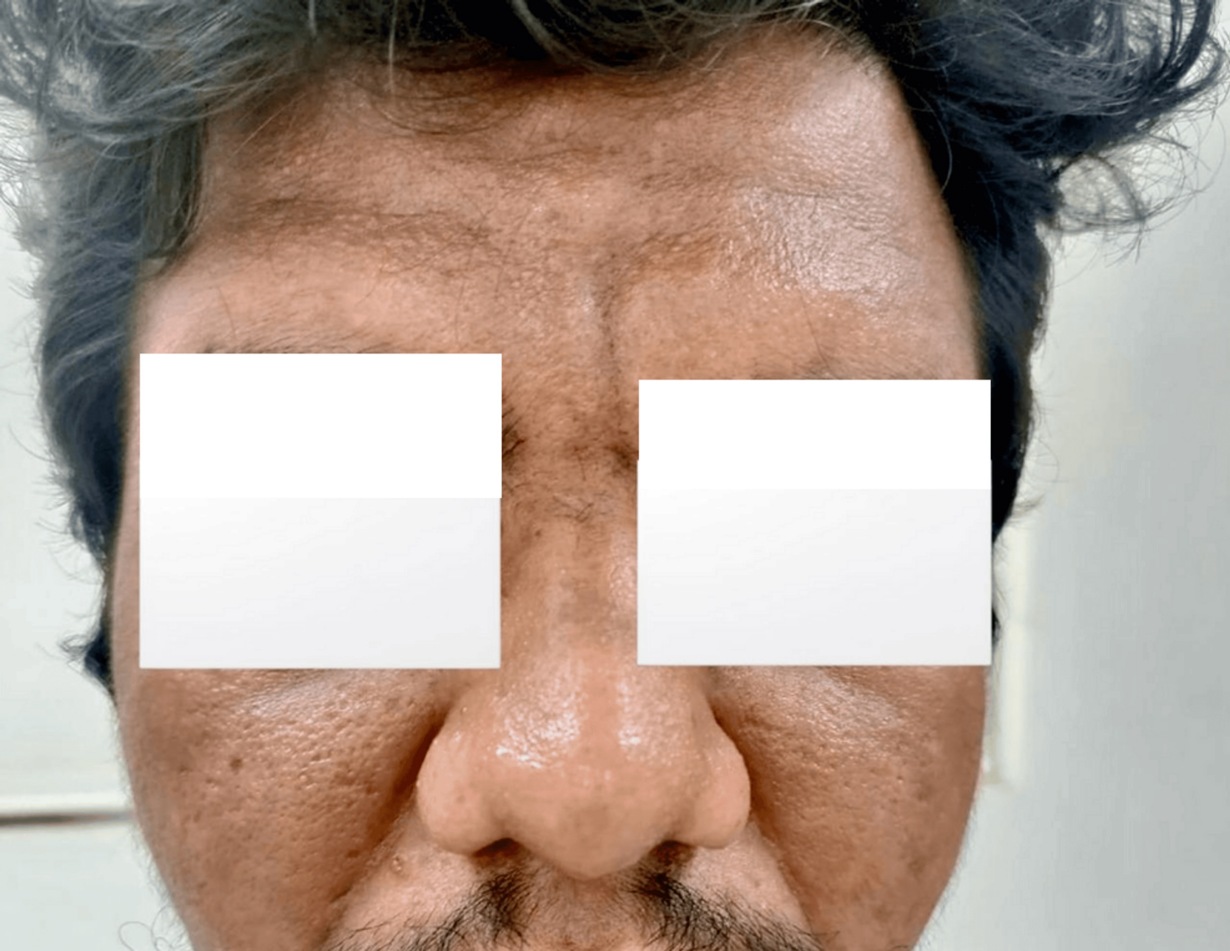
Pachydermia of the face, prominent forehead wrinkles, and seborrhoea.

**Figure 2:**
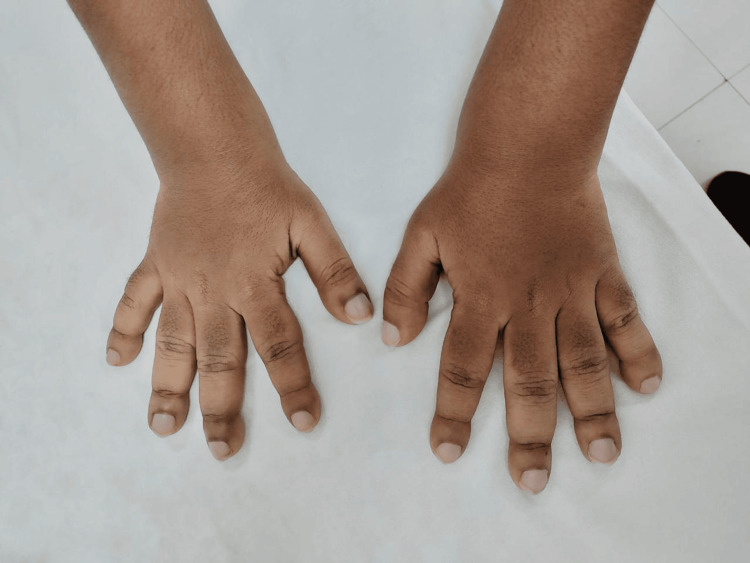
Digital clubbing of the upper limbs.

**Figure 3:**
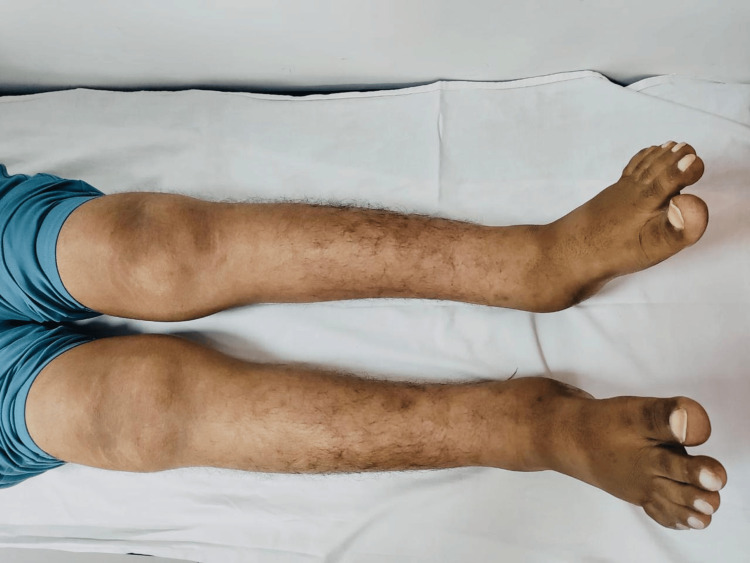
Digital clubbing of the lower limbs.

On physical examination, the patient presented with several distinctive features. His face had a coarse appearance, with deep furrows on his forehead (Figure 1). His hands and feet showed severe digital clubbing, with bulbous enlargement of the fingertips and toes (Figures 2, 3). The skin on his face and extremities was noticeably thickened and oily, and he reported excessive sweating (hyperhidrosis), which added to his discomfort. The joint pain predominantly affected the wrists, ankles, and knees, with intermittent swelling and stiffness but no erythema or warmth. On palpation, there was diffuse tenderness along the distal radius and tibia, suggestive of periosteal involvement. No other family members reported similar findings.

The patient was managed conservatively with NSAIDs, emollients for seborrhea, and advised on joint physiotherapy and stress reduction. After six months of follow-up, he reported partial relief of pain and improvement in joint stiffness.

**Figure 4:**
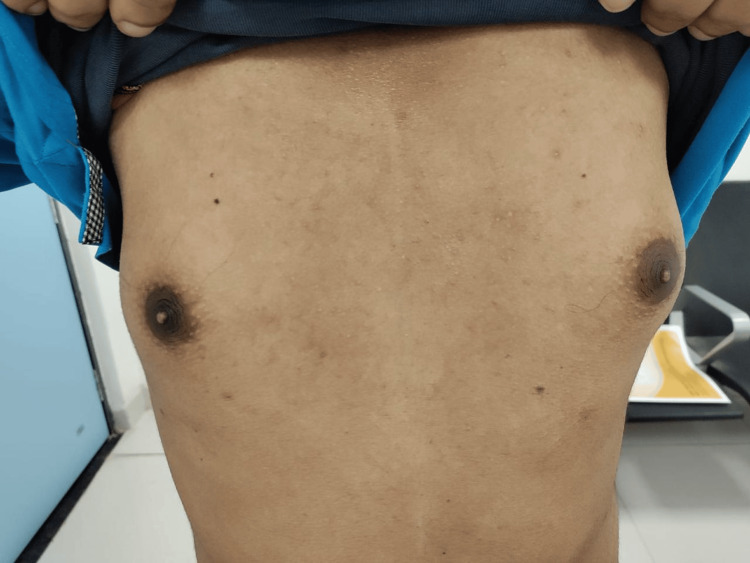
Bilateral gynecomastia.

A particularly intriguing finding was the presence of bilateral gynecomastia, an enlargement of the male breast tissue, which is not commonly associated with pachydermoperiostosis (Figure 4). His vital signs were stable, and a systemic examination revealed no abnormalities in the cardiovascular, respiratory, or abdominal systems. The presence of gynecomastia in this patient may be related to altered prostaglandin metabolism, which can influence local estrogen-androgen balance and peripheral aromatization. Similar reports suggest that secondary endocrine dysregulation involving the growth hormone-insulin-like growth factor-1 (GH-IGF-1) axis may occur in pachydermoperiostosis, and gynecomastia may represent a rare endocrine manifestation rather than a coincidental finding [3,8,9].

To rule out secondary causes of his symptoms, a series of investigations was performed. Routine laboratory tests, including a complete blood count, renal function tests, liver function tests, hormonal assays, thyroid function tests, etc., were all within normal limits. Given the patient’s coarse facial features and enlarged extremities, acromegaly was considered a potential differential diagnosis. However, his IGF-1 (somatomedin-C) levels were found to be low, which ruled out acromegaly and raised questions about potential GH axis abnormalities [10]. The hormonal test results are shown in Table 1.

**Table 1:** Laboratory investigations

Test	Patient Value	Reference Range
Insulin-like Growth Factor-1 (IGF-1)	110 ng/mL	117–323 ng/mL
Cortisol (Morning Sample)	12.3 µg/dL	5–25 µg/dL
Luteinizing Hormone (LH)	4.2 mIU/mL	1.7–8.6 mIU/mL
Testosterone	520 ng/dL	300–1000 ng/dL
Prolactin	8.5 ng/mL	2–18 ng/mL
Adrenocorticotropic Hormone (ACTH)	23 pg/mL	7–63 pg/mL
Growth Hormone	0.4 ng/mL	<3 ng/mL
Thyroid Stimulating Hormone (TSH)	2.5 µIU/mL	0.4–4.2 µIU/mL
Triiodothyronine (T3)	120 ng/dL	80–200 ng/dL
Thyroxine (T4)	8.6 µg/dL	5.1–14.1 µg/dL

**Figure 5:**
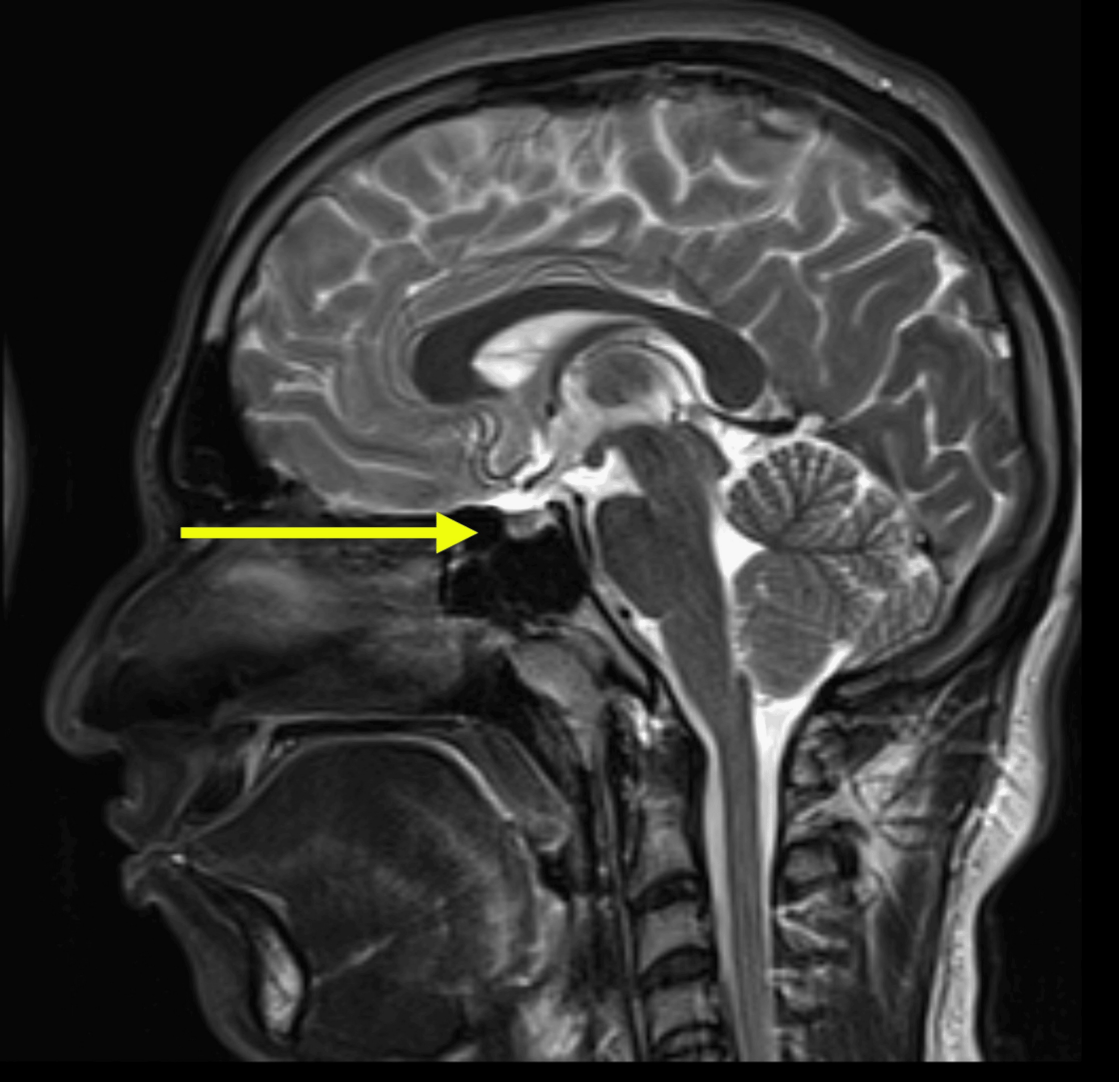
Sagittal T2‑weighted MRI of the brain showing the pituitary gland (yellow arrow) within the sella turcica. The pituitary stalk and optic chiasma are well visualised, with no abnormal signal changes or mass effect.

**Figure 6:**
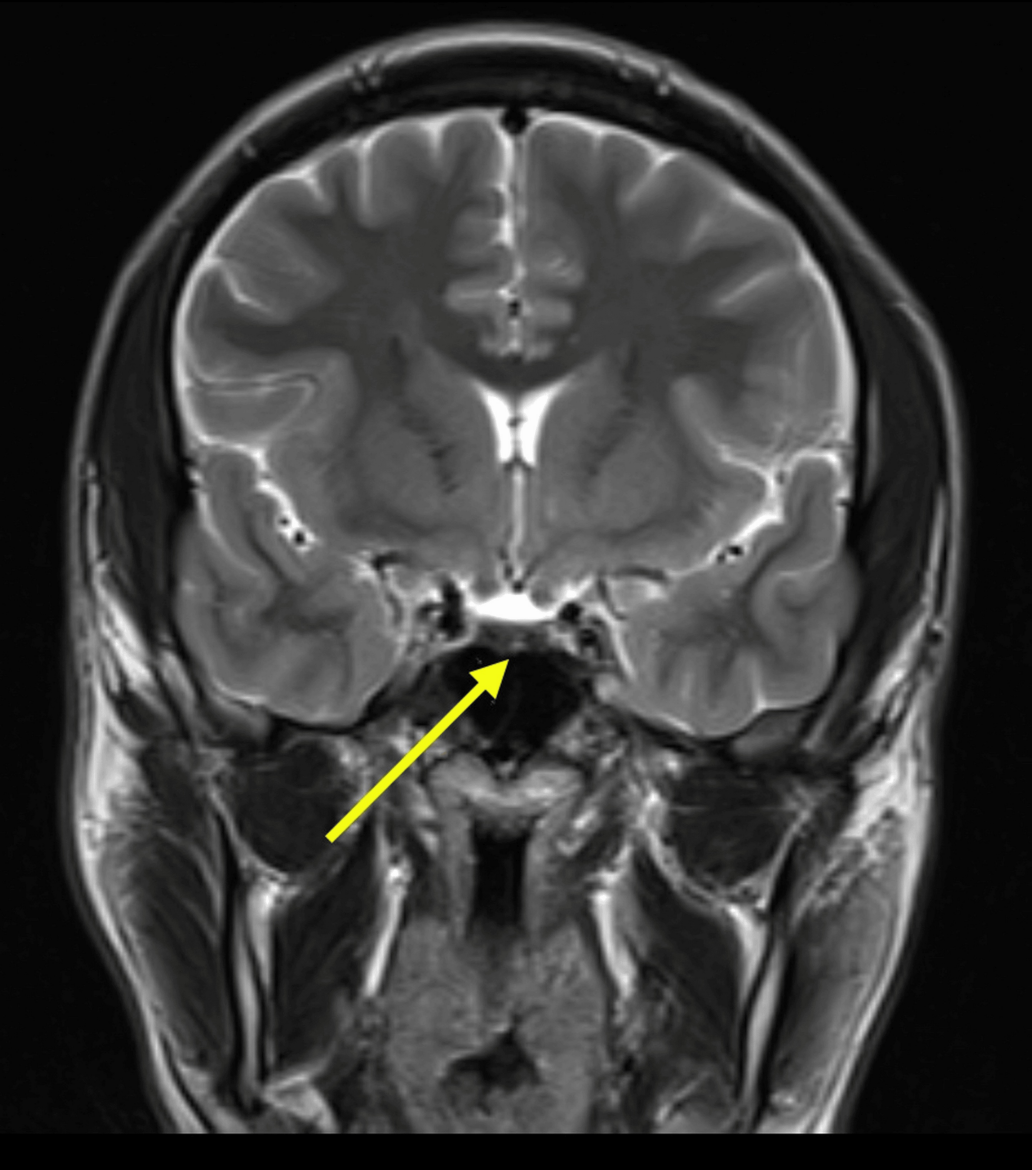
Coronal T2‑weighted MRI of the brain demonstrating the sella turcica and pituitary gland (yellow arrow) with normal size, morphology, and signal intensity. No evidence of focal lesions or suprasellar extension is seen.

The clinical findings of digital clubbing, pachydermia, and periostosis (based on physical examination) were highly suggestive of pachydermoperiostosis. Although genetic testing for mutations in the *HPGD* or *SLCO2A1* genes would have confirmed the diagnosis, it was also not performed due to resource constraints. An MRI of the brain and pituitary was performed to exclude any structural abnormalities or tumors that could explain his symptoms, but the results were unremarkable (Figures 5, 6). Nevertheless, the combination of clinical features and family history was sufficient to establish a diagnosis of pachydermoperiostosis.

## Discussion

PDP, also known as primary hypertrophic osteoarthropathy, is a rare genetic disorder characterized by digital clubbing, pachydermia (skin thickening), and periostosis. It is an autosomal dominant condition with variable penetrance, typically manifesting during adolescence and progressing over several years before stabilizing [4]. Unlike secondary hypertrophic osteoarthropathy, PDP occurs without an underlying pulmonary, cardiac, or hepatic disease.

Our patient, a 20-year-old male, presented with digital clubbing, coarse facial features, gynecomastia, and joint pain. Laboratory evaluation revealed low IGF-1 (somatomedin-C) levels, raising the initial concern for growth hormone deficiency or pituitary dysfunction. However, MRI of the brain and pituitary fossa was normal, ruling out structural abnormalities or pituitary adenomas. These findings support previous studies that have reported normal or low IGF-1 levels in PDP, distinguishing it from acromegaly, where IGF-1 is typically elevated due to excess growth hormone secretion [11]. Interestingly, the presence of gynecomastia in our patient adds an uncommon but previously reported feature of PDP [9]. Further research is needed to determine whether gynecomastia is an incidental finding or part of the phenotypic spectrum of PDP.

Radiographic confirmation of periosteal thickening is considered a hallmark of PDP. However, in our case, X-rays were not performed, as the diagnosis was made based on strong clinical findings and the exclusion of secondary causes. While imaging remains useful for diagnostic confirmation, cases with a clear clinical presentation may not necessarily require radiographic evaluation for diagnosis [12].

Given the progressive but non-life-threatening nature of PDP, treatment remains largely symptomatic, focusing on pain relief (NSAIDs), dermatological care, and orthopedic support [13]. This case underscores the clinical heterogeneity of PDP, particularly in relation to endocrine involvement. The presence of low IGF-1 levels, normal pituitary imaging, and gynecomastia highlights the need for individualized evaluation rather than reliance on classical diagnostic criteria. Early recognition and a multidisciplinary management approach can significantly improve patient outcomes and quality of life. 

Although clinical findings strongly suggested PDP, the absence of radiographic confirmation of periostosis and lack of genetic testing prevent definitive confirmation. Therefore, this case should be considered a probable diagnosis of PDP, established through major clinical criteria and exclusion of secondary causes.

## Conclusions

PDP is a rare disorder with diverse clinical manifestations. This case represents a probable diagnosis of PDP with rare endocrine involvement presenting as gynecomastia and low IGF-1 levels. Our case highlights the importance of considering PDP in young men presenting with digital clubbing and associated features such as gynecomastia. The low IGF-1 levels in our patient, despite normal pituitary imaging, emphasize the need for careful differentiation from acromegaly. Additionally, the absence of radiographic evaluation did not preclude diagnosis, reinforcing that strong clinical features can be diagnostic in certain cases. While PDP is not life-threatening, its progressive nature necessitates early diagnosis and symptomatic management to improve long-term quality of life.
